# Uncovering the heterogeneity of NK cells on the prognosis of HCC by integrating bulk and single-cell RNA-seq data

**DOI:** 10.3389/fonc.2025.1570647

**Published:** 2025-03-18

**Authors:** Jiashuo Li, Zhenyi Liu, Gongming Zhang, Xue Yin, Xiaoxue Yuan, Wen Xie, Xiaoyan Ding

**Affiliations:** ^1^ National Center for Infectious Diseases, Beijing Di’tan Hospital, Capital Medical University, Beijing, China; ^2^ Department of Interventional Radiology, Beijing Friendship Hospital, Capital Medical University, Beijing, China; ^3^ Department of General Surgery, Beijing You’an Hospital, Capital Medical University, Beijing, China; ^4^ Cancer Center, Beijing Di’tan Hospital, Capital Medical University, Beijing, China

**Keywords:** hepatocellular carcinoma, natural kill cell, tumor microenvironment, nomogram, prognosis, TUBA1B, single-cell

## Abstract

**Background:**

The tumor microenvironment (TME) plays a critical role in the development, progression, and clinical outcomes of hepatocellular carcinoma (HCC). Despite the critical role of natural killer (NK) cells in tumor immunity, there is limited research on their status within the tumor microenvironment of HCC. In this study, single-cell RNA sequencing (scRNA-seq) analysis of HCC datasets was performed to identify potential biomarkers and investigate the involvement of natural killer (NK) cells in the TME.

**Methods:**

Single-cell RNA sequencing (scRNA-seq) data were extracted from the GSE149614 dataset and processed for quality control using the “Seurat” package. HCC subtypes from the TCGA dataset were classified through consensus clustering based on differentially expressed genes (DEGs). Weighted gene co-expression network analysis (WGCNA) was employed to construct co-expression networks. Furthermore, univariate and multivariate Cox regression analyses were conducted to identify variables linked to overall survival. The single-sample gene set enrichment analysis (ssGSEA) was used to analyze immune cells and the screened genes.

**Result:**

A total of 715 DEGs from GSE149614 and 864 DEGs from TCGA were identified, with 25 overlapping DEGs found between the two datasets. A prognostic risk score model based on two genes was then established. Significant differences in immune cell infiltration were observed between high-risk and low-risk groups. Immunohistochemistry showed that HRG expression was decreased in HCC compared to normal tissues, whereas TUBA1B expression was elevated in HCC.

**Conclusion:**

Our study identified a two-gene prognostic signature based on NK cell markers and highlighted their role in the TME, which may offer novel insights in immunotherapy strategies. Additionally, we developed an accurate and reliable prognostic model, combining clinical factors to aid clinicians in decision-making.

## 1 Introduction

Primary liver cancer is one of the most common malignant tumors, the sixth most prevalent cancer in the world, and the second leading cause of cancer-related deaths (1, 2). Hepatocellular carcinoma is the predominant type of liver cancer (80%-90%). The majority of patients with HCC are diagnosed during the late stages of the disease. Clinical benefit is limited in patients with advanced HCC, with a median overall survival (OS) of only 1-1.5 years (3, 4). In terms of treatment, the primary methods for liver cancer treatment include surgical resection, local ablation, transarterial chemoembolization (TACE), and liver transplantation. In recent years, immunotherapy has also shown potential in the treatment of HCC, providing patients with new treatment options. In addition, immune-related genes play an important role in tumour immunotherapy. Tumor microenvironment (TME) refers to the surrounding environment in which tumor cells exist (5, 6). Nowadays, it is widely accepted that the components of the TME, such as immune cells and inflammatory cells, play a pivotal role in tumor development, progression, and clinical outcomes (7–9). Consequently, alterations in the TME have an impact on patient prognosis, making it critical to identify TME biomarkers to predict patient outcomes (10, 11).

Natural killer (NK) cells are cytotoxic lymphocytes and components of innate immunity, capable of recognizing and eliminating damaged or stressed cells (12). In the liver, NK cells make up 30-50% of intrahepatic lymphocytes (13). Immune cells are able to directly kill tumor cells and promote T-cell immune responses in tumor immunity, thereby inhibiting the occurrence and development of cancer (14, 15). In many types of cancer, the infiltration level of NK cells is reduced, including gastric, esophageal, breast, and colon cancers (16–18). However, studies on NK cells in patients with HCC are scarce. Moreover, high levels of infiltrated NK cells in tumor tissues are associated with better prognosis in patients with cancer (19, 20). Therefore, in-depth characterization of NK cell phenotypes and functionality is crucial for understanding liver cancer and could help determine therapeutic strategies for HCC patients.

Traditional bulk RNA sequencing (bulk RNA-seq) averages the transcriptional profiles of cells in one sample, which is strongly influenced by cell type dominance. In addition, bulk RNA-seq is unable to effectively distinguish between different cell lineages and cellular interactions. The advent of single-cell mRNA sequencing (scRNA-seq) has broadened the understanding of cellular components and gene expression specificities in the TME (21). Single-cell sequencing is a technique that allows the analysis of gene expression at the individual cell level. It involves isolating single cells, amplifying their RNA or DNA, and sequencing the genetic material to uncover cellular heterogeneity and molecular profiles. ScRNA-seq highlight heterogeneity and distinct subpopulations within tumors, allowing for enumeration and quantification of immune infiltration in tumor tissue (22, 23). The heterogeneous immune cell infiltrates are a crucial factor for treatment response and prognosis in HCC and other tumor types (24–26). The majority of scRNA-seq studies on immune cells derived from HCC tissues have predominantly focused on T cell characteristics, with limited research dedicated to NK cells (25, 27). Consequently, there is an urgent need for scRNA-seq analysis of NK cells from both healthy liver and HCC tissues to uncover NK cell-related prognostic genes that contribute to a deeper understanding of HCC prognosis.

In this study, different cell subsets between tumor tissues and normal control tissues were identified from the HCC single-cell dataset of the Gene Expression Omnibus (GEO) database. In conjunction with bulk RNA-seq analysis of The Cancer Genome Atlas (TCGA) cohort and the International Cancer Genome Consortium (ICGC) cohort, NK cell marker gene-related features for predicting HCC prognosis were created, and a model was built with clinical indicators and NK cell risk profiles. Our study expanded the exploration of HCC and contributed novel insights into HCC diagnosis, therapies, and prognosis.

## 2 Methods and materials

### 2.1 HCC data and processing in public databases

The gene expression dataset (GSE149614) consists of single-cell samples from 10 HCC patients, including ten primary tumor samples and eight adjacent normal samples. The raw data contained a total of 25479 genes and 71915 cells. The Seurat function from the R package was employed to ensure each gene was expressed in a minimum of 3 cells and each cell had at least 250 genes expressed to filter single cells. The percentage of mitochondria and rRNA quantities were calculated using the “PercentageFeatureSet” function. Cells with less than 200 genes, a percentage of mitochondrial reads less than 25%, and a median UMI count of less than 500 were excluded from the downstream analysis. After filtering, 53293 cells were retained.

The RNA-seq transcriptome information and matching clinical data were downloaded from the TCGA database (https://portal.gdc.cancer.gov/) and ICGC database (https://dcc.icgc.org/).

### 2.2 Single-cell RNA sequencing data analysis

The “SCTransform” method was adopted for data normalization, and the “FindVariableFeatures” function was applied to identify the top 2000 highly variable genes (HVGs) (28). The integrated data were scaled by the “Sclae Data” function, and principal component analysis (PCA) was performed using the “Run PCA” function for 2000 HVGs. The “FindNeighbors” and “FindClusters” functions were implemented to find cell clusters when dim=50 and resolution=0.4 (29, 30). Next, we selected the top 20 principal components in order to further reduce the dimensionality using the UMAP method. UMAP is a method of data dimensionality reduction, which assumes that the available data samples are uniformly distributed in a topological space (Manifold), and these limited data samples can be approximated (Approximation) and mapped (Projection) to a lower dimensional space. Differential genes (DEGs) in different cell types were identified by setting logfc=0.5, minpct=0.25, and adjusting P<0.05 in Seurat’s Findallmarker function. Cell cluster annotation was based on marker genes obtained from the literature and the Cellmarker Database.

### 2.3 Differential gene expression analysis

DEGs in the dataset GSE149614 were identified and confirmed using the limma package, which applies linear models to detect genes that show significant changes in expression across different experimental conditions. In the TCGA dataset, the limma, DESeq2, and edgeR packages were utilized. Each of these tools employs different statistical approaches for identifying DEGs, such as linear models, data normalization and dispersion estimation, and negative binomial distribution for RNA-seq count data. By using these methods, we ensured a comprehensive and reliable analysis of the differentially expressed genes across the dataset, leading to more accurate results.

### 2.4 Clustering

The DEGs in the TCGA-LIHC obtained by the three algorithms were taken to be intersected. We determined a total of 356 up-regulated and 168 down-regulated genes. Consistent clustering analysis was performed in TCGA-LIHC samples by using the “ConsensusClusterPlus” R package to identify molecular subtypes. Pam arithmetic and “Spearman” distance were utilized to complete 500 bootstraps, with every bootstrap having specimens (≥80%) of the TCGA-LIHC dataset. Cluster number k was between 2 and 10, and optimal clusters were screened by cumulative distribution function (CDF) curve and consensus CDF. Survival differences among the molecular subtypes were estimated by Kaplan-Meier (K-M) curves using the log-rank test. Additionally, differences in the distribution of clinical characteristics between molecular subtypes were compared with chi-square tests.

### 2.5 Weighted gene co-expression network analysis

The gene expression data profiles of TCGA were constructed for gene co-expression networks using the WCGNA package in R, including module identification, network generation, gene screening, calculation of properties, and data visualization. Correlations between gene pairs were first calculated using gene expression profiling and transformed into a collocation matrix. Then, the soft threshold was set to make network construction among the genes in the network obey scale-free networks, and the adjacency matrix was transformed into a topological overlap matrix (TOM). Branches of the cluster tree and different colors represent different gene modules. The correlation between module eigengenes and clinical traits was assessed using the Pearson correlation test to identify the significant modules.

### 2.6 Construction of a prognostic risk model for HCC

By analyzing the intersection of prognosis-related genes and DEGs, the death differential genes related to the prognosis of HCC were finally obtained (31). Univariate and multivariate COX analyses were employed to screen for genes with P<0.05 as prognostically relevant genes for HCC and to establish risk regression scores. According to the predictive risk scores, patients were divided into high and low-risk groups to explore the survival difference (32).

### 2.7 Development of the nomogram

After combining risk scores and clinical factors, a nomogram was established. We can accurately predict 1-, 3-, and 5-year survival in HCC patients by calculating cumulative scores based on individual factors. The predictive ability and accuracy of the nomogram were assessed by the ROC curve, calibration curve, and DCA curve.

### 2.8 Immune cell infiltration and correlation analysis

The relative infiltration of immune cell types in the tumor microenvironment was assessed by Single Sample Gene Set Enrichment Analysis (ssGSEA), including monocyte, central memory CD4 T cell, CD56dim natural killer cell, plasmacytoid dendritic cell, central memory CD8 T cell, immature dendritic cell, natural killer cell, activated dendritic cell, gamma delta T cell, CD56bright natural killer cell, memory B cell, MDSC, T follicular helper cell, activated CD8 T cell, Effector memory CD8 T cell, Type 1 T helper cell, Type T helper cell, natural killer T cell, regulatory T cell, effector memory CD4 T cell, activated CD4 T cell, Type 17 T helper cell, macrophage, immature B cell, mast cell, eosinophil, neutrophil, and activated B cell. Expression data were used to further analyze the correlation between the screened genes and immune cells.

### 2.9 Immunohistochemical staining

For IHC staining, HCC tissues were fixed in 4% paraformaldehyde and embedded in paraffin blocks, followed by a process of dewaxing and rehydration (33, 34). Peroxidase activity was blocked with 3% hydrogen peroxide. Sections were incubated overnight at four°C with primary antibodies (anti-HRG, anti-TUBAB1) purchased from Abcam. The tissue sections were treated with biotinylated secondary antibody, then stained with diaminobenzidine substrate-chromogen solution, and counterstained with hematoxylin. The images were captured in the XSP-CD204 microscope. It helps validate the expression, localization, and distribution of proteins within tissues, providing insight into the molecular characteristics of diseases, such as cancer, and confirming the presence of specific biomarkers for diagnostic or research purposes.

### 2.10 Statistical analysis

All statistical analyses were conducted using R software (version 4.1.3). Continuous variables were compared using the Mann-Whitney U or Kruskal-Wallis test, and categorical variables were compared using the Chi-square test or Fisher’s Exact test. Correlations were examined using Spearman rank analysis. Statistical significance was claimed for P<0.05 (two-sided).

## 3 Result

### 3.1 Identification of tumor-associated NK cell marker genes

**Figure 1:**
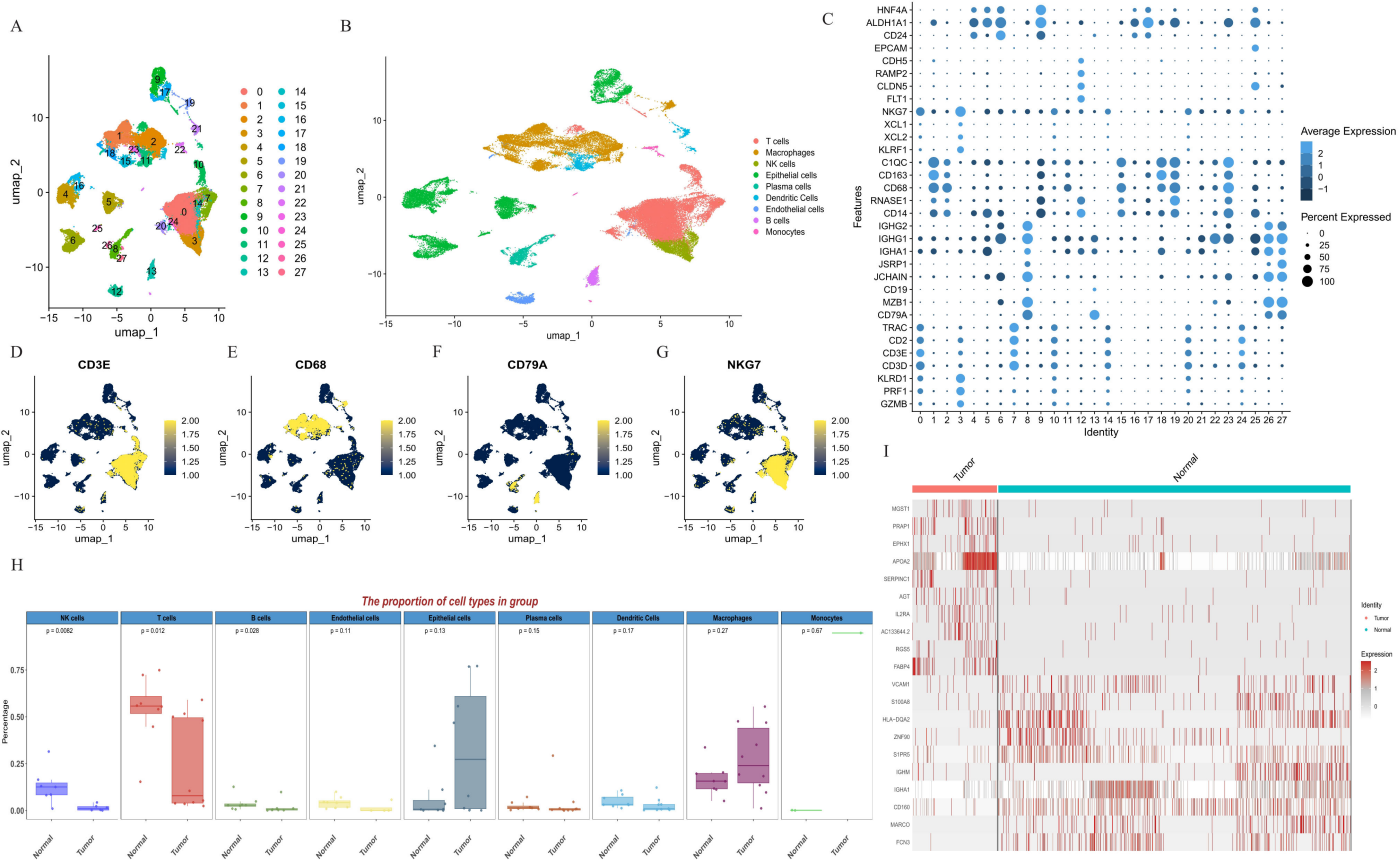
Definition of cell clusters. **(A)** U-MAP of 27 cell subgroups. **(B)** U-MAP of nine cell types. **(C)** The expression of major marker genes in 27 cell clusters. **(D)** The expression of CD3E in the 27 clusters. **(E)** The expression of CD68 in the 27 clusters. **(F)** The expression of CD79A in the 27 clusters. **(G)** The expression of NKG7 in the 27 clusters. **(H)** The proportion of cell types in tumor tissues and para-cancer tissues. **(I)** Differential genes associated with NK cells. NK cells, natural killer cells; U-MAP, uniform manifold approximation and projection.

By executing the Seurat function and PercentageFeatureSet function, a total of 53293 cells were screened in the scRNA GSE149614 dataset. The quality control before and after filtration was displayed (**Supplementary Figure S1**). After log-normalization and dimensionality reduction of the data, we obtained the distribution of the nine cell clusters by UMAP visualization (**Figures 1A, B**). The results of the significant genes with the top20 in rank change values are list in **Supplementary Table S1**. These cells were manually annotated by cell type based on marker expression, involving NK cells (NKG7, KLRD1), Macrophages (CD68), Epithelial cells, plasma cells (CD138), T cells (CD3D+, CD3E), Endothelial cells (CD31), and B cells (CD79A, MS4A6A), Dendritic Cells (CD141), monocytes (CD14) (**Figures 1C–G**, **Supplementary Figure S2**). Comparative the tumor samples from patients HCC01, HCC02, HCC03, HCC04, HCC05, HCC06, HCC07, HCC08, HCC09, HCC10 and adjacent non-tumor tissues from HCC03, HCC04, HCC05, HCC06, HCC07, HCC08, HCC09, HCC 10, it was found that the proportion of NK cells in the tumor tissues was significantly lower than in the adjacent non-tumor tissues (**Figure 1H**). **Figure 1I** demonstrated the DEGs between the tumor and para-cancer in NK cells.

### 3.2 Consensus clustering identified two clusters

**Figure 2:**
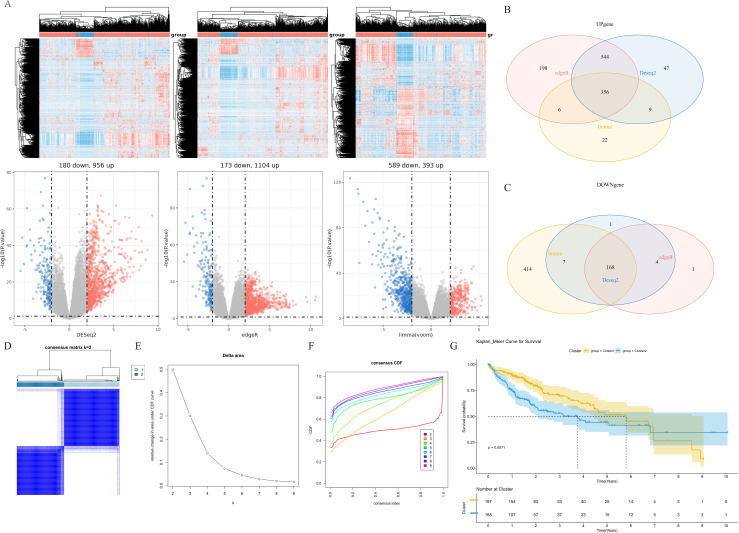
Identification of molecular subtypes. **(A)** Differential genes associated with different groups in TCGA cohort. **(B)** Venn diagram of upregulated differential genes. **(C)** Venn diagram of downregulated differential genes. **(D)** Heatmap of sample clustering when K=2. **(E)** Delta area. **(F)** Cumulative distribution function. **(G)** K-M survival analysis of cluster 1 and cluster 2 in the TCGA-LIHC dataset. TCGA, The Cancer Genome Atlas; K-M, Kaplan-Meier.

To investigate the cause of hepatocellular carcinoma development at the molecular level, we performed differential analysis by calling the dataset from the TCGA database and using the Deseq2 package, edgeR package, and limma package (**Figure 2A**). The common set of differentially expressed genes is represented by the intersection of the three sets of genes, including 356 up-regulated and 168 down-regulated genes (524 genes in total, **Figures 2B, C**, **Supplementary Table S2**). Based on the expression of these 524 DEGs, we clustered the 360 samples from TCGA-LIHC using the ConsencusClusterPlus package, and according to the cumulative distribution function (CDF) and incremental area, two clusters (Cluster 1 and Cluster 2) were obtained when K=2 (**Figure 2D–F**, **Supplementary Figure S3**). K-M analysis revealed that patients in Cluster 2 had a markedly worse overall survival (OS) than patients in Cluster 1 (**Figure 2G**).

### 3.3 Prognostic genes were screened out by WCGNA

**Figure 3:**
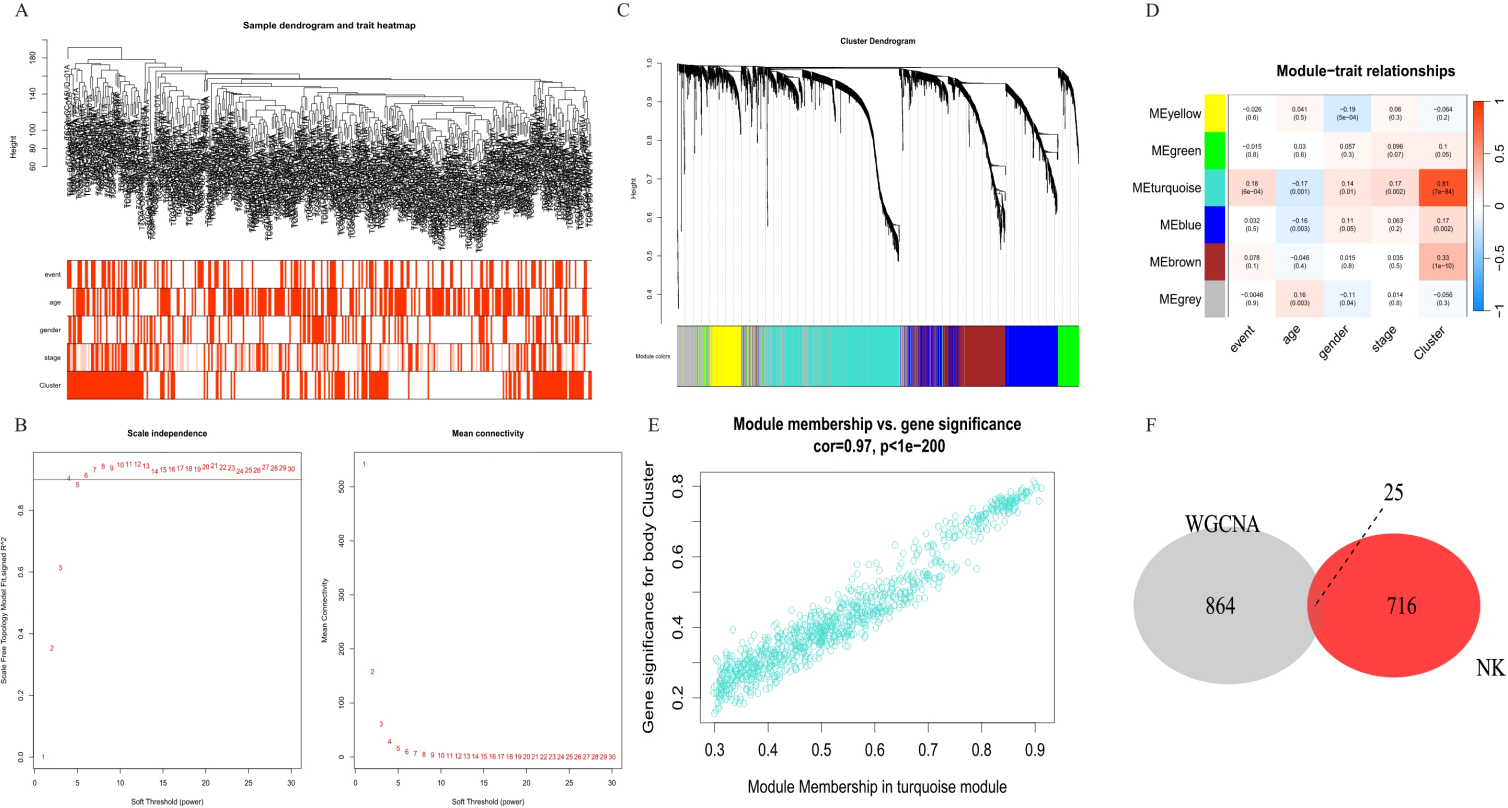
Identification of co-expression modules in HCC. **(A)** Sample clustering to detect outliers. **(B)** The scale-free fit index for soft-thresholding powers. **(C)** Constructing a gene dendrogram based on different metrics. **(D)** Heatmap of the correlation between 6 modules and clinical characteristics. **(E)** The correlation between turquoise module and prognostic cluster. **(F)** Venn diagram of NK cell differential genes and turquoise module. NK cells, natural killer cells; HCC, hepatocellular carcinoma.

To further analyze the correlation between gene expression patterns and distinct cell subgroups in HCC, we performed the WGCNA method to construct key modules based on the two clusters, and the results of the hierarchical cluster analysis of all samples are presented in **Figure 3**. We constructed a co-expression network in HCC by calculating the Pearson correlation coefficient in the two clusters based on the WCGNA analysis (**Figure 3D**). The Pearson correlation test was used to assess the relationship between module eigengenes and clinical traits, helping to identify the significant modules. Modules with a p-value less than 0.05 were considered significant. The hierarchical clustering was applied to cluster genes, and a total of 6 modules were obtained. Among them, the turquoise module was the most relevant module with a prognosis (P<1e-200, **Figure 3E**). Then, we selected the intersection of highly expressed genes in NK cell lines and genes in the turquoise module in WCGNA analysis for subsequent analysis (**Figure 3F**). A total of 25 prognosis-related candidate genes were identified.

### 3.4 Identification of prognosis-related genes

**Figure 4:**
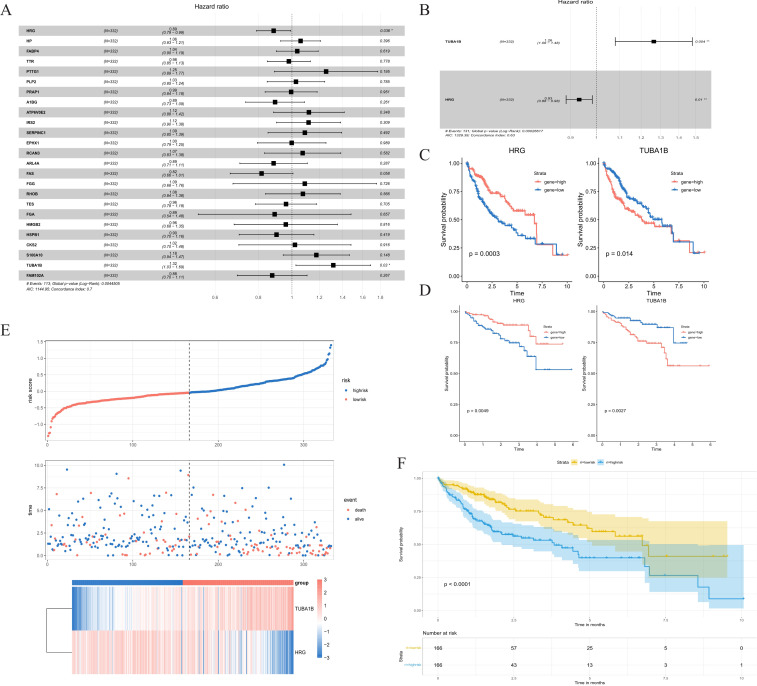
NK cell signature establishment. **(A)** Univariate Cox analysis of screened genes. **(B)** Multivariate Cox analysis of screened genes. **(C)** K-M curve compares the overall HCC patients in the TCGA cohort. **(D)** K-M curve compares the overall HCC patients in the ICGC cohort. **(E)** Distribution of risk scores and patient survival between low and high-risk groups in the TCGA cohort. **(F)** KM curve compares the overall HCC patients between high-risk and low-risk groups in the TCGA cohort. NK cells, natural killer cells; K-M, Kaplan-Meier; TCGA, The Cancer Genome Atlas.

HRG and TUBAIB were screened as prognostically relevant genes for HCC by univariate and multivariate COX regression analysis (**Figures 4A, B**), and the risk score model was established (**Figure 4E**). Results at both TCGA and ICGC cohorts showed that HRG gene indicated association with better prognosis while TUBA1B indicates association with poor prognosis (**Figures 4C, D**, **Supplementary Figure S4**). Patients were assorted into high-risk and low-risk groups in accordance with the median value of risk scores. Survival analysis exhibited significant discrimination in OS among the groups (P<0.001, **Figure 4F**).

### 3.5 Development of the nomogram combining risk score and clinicopathological indicators in TCGA-LIHC

**Figure 5:**
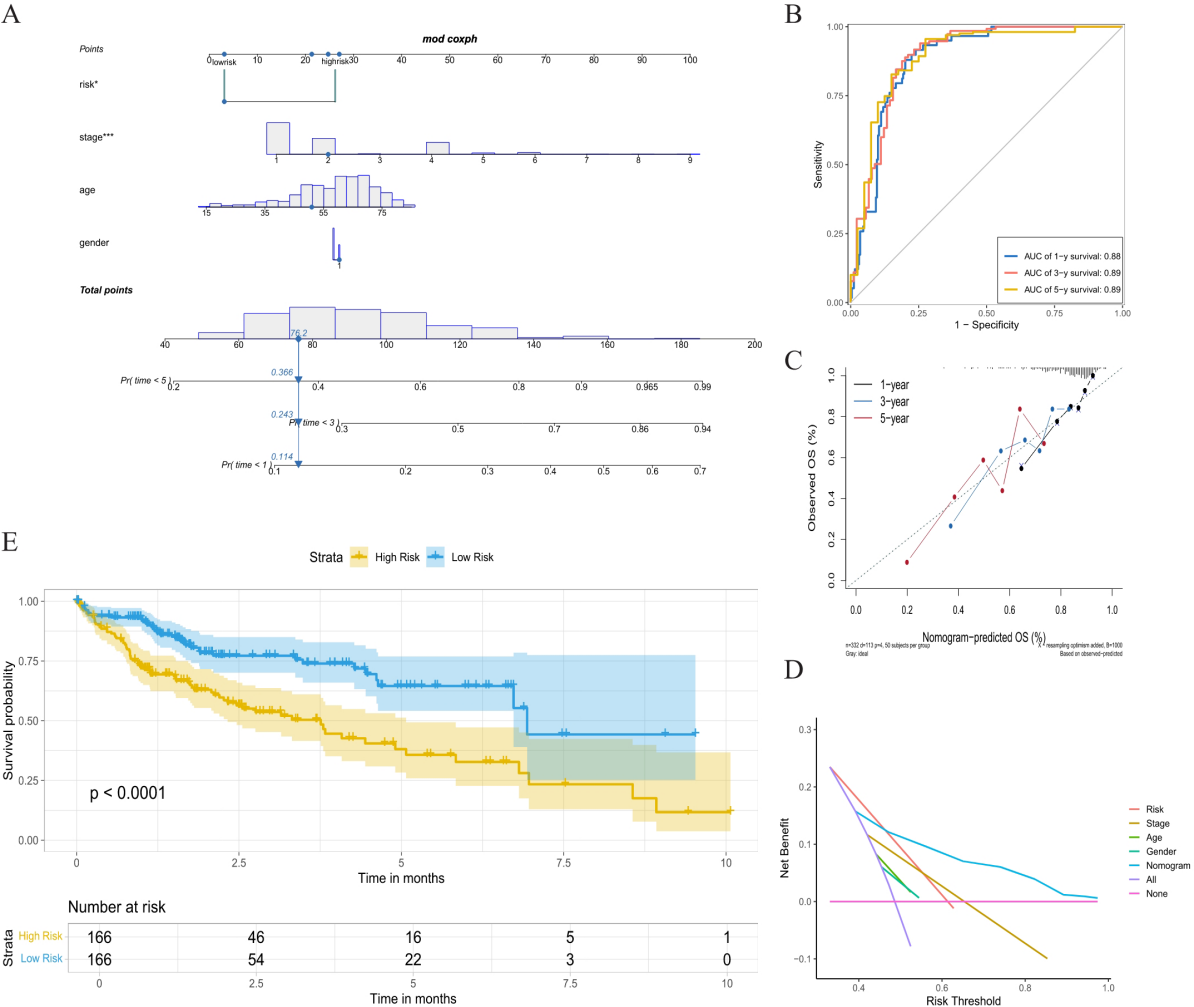
Nomogram analysis based on multivariate Cox regression. **(A)** Nomogram combing the risk score, stage, age, and gender. **(B)** Time-dependent ROC curves analysis of 1-year, 3-year, and 5-year survival. **(C)** The calibration curve of 1-year, 3-year, and 5-year survival. **(D)** Decision curve analysis. **(E)** K-M curve between high-risk group and low-risk group. ROC, receiver operating characteristic; K-M, Kaplan-Meier.

We subsequently examined the association between risk scores and clinical variables, which were statistically different for group, event, and gender. The nomogram was constructed by risk score, age, stage, and gender (**Figure 5A**). The ROC curves produced the 1-, 3-, and 5-year area under the curve (AUCs) were 0.88, 0.89, and 0.89 (**Figure 5B**). The calibration curve showed that the predicted value of the nomogram was in good agreement with the actual observed value, and the DCA curve showed that the nomogram had a better clinical net benefit and better clinical applicability (**Figures 5C, D**). Patients were classified into low-risk and high-risk groups according to a cutoff value of 50% predicted by the nomogram. The KM curves demonstrated significantly higher OS with the low-risk group (**Figure 5E**).

### 3.6 Immune landscape based on risk score

**Figure 6:**
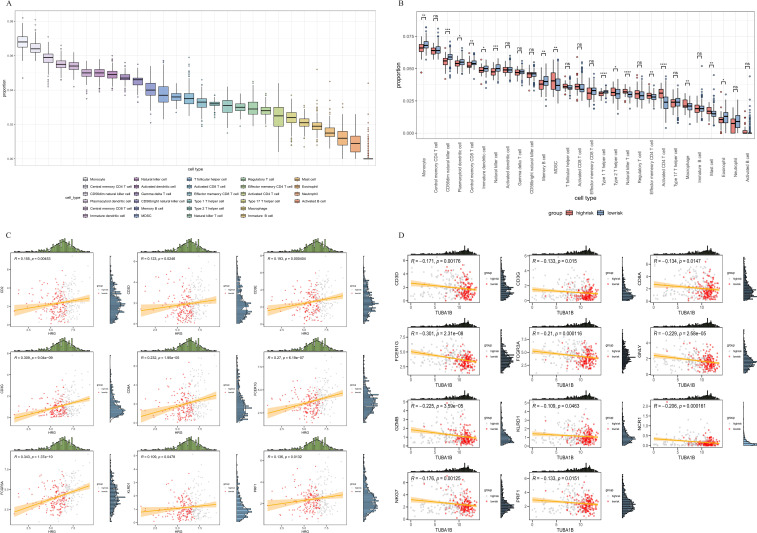
Correlation analysis between the signature and immunity. **(A)** Immune cell proportion of all tissues in the TCGA-LIHC cohort. **(B)** Immune cell proportion of high-risk and low-risk groups in the TCGA-LIHC cohort. **(C, D)** Correlation of immune cell infiltration and risk score. TCGA, The Cancer Genome Atlas.

We investigated the proportions of various immune cell types in the HCC sample and para-cancer sample (**Figure 6A**). The considerable variations in immune cell infiltration between the high- and low-risk groups were evident from the single sample GSEA (ssGSEA). The results displayed that the proportion of monocytes, CD56dim NK cells, CD56bright NK cells, and CD8+ T cells was higher in the low-risk group than in the high-risk group (P<0.05). The CD4+ T cell, MDSC, macrophages, and mast cells of the high-risk group were substantially elevated compared with that of the low-risk group (**Figure 6B**). Correlation analysis showed that HRG was positively correlated with increased NK cells, and TUBA1B was negatively correlated with increased NK cells (**Supplementary Figures S5C, D**). We further confirmed the results by correlation analyses of immune cells and immune molecules (**Figures 6C, D**, **Supplementary Figure S5**).

### 3.7 Validation of prognostic genes

**Figure 7:**
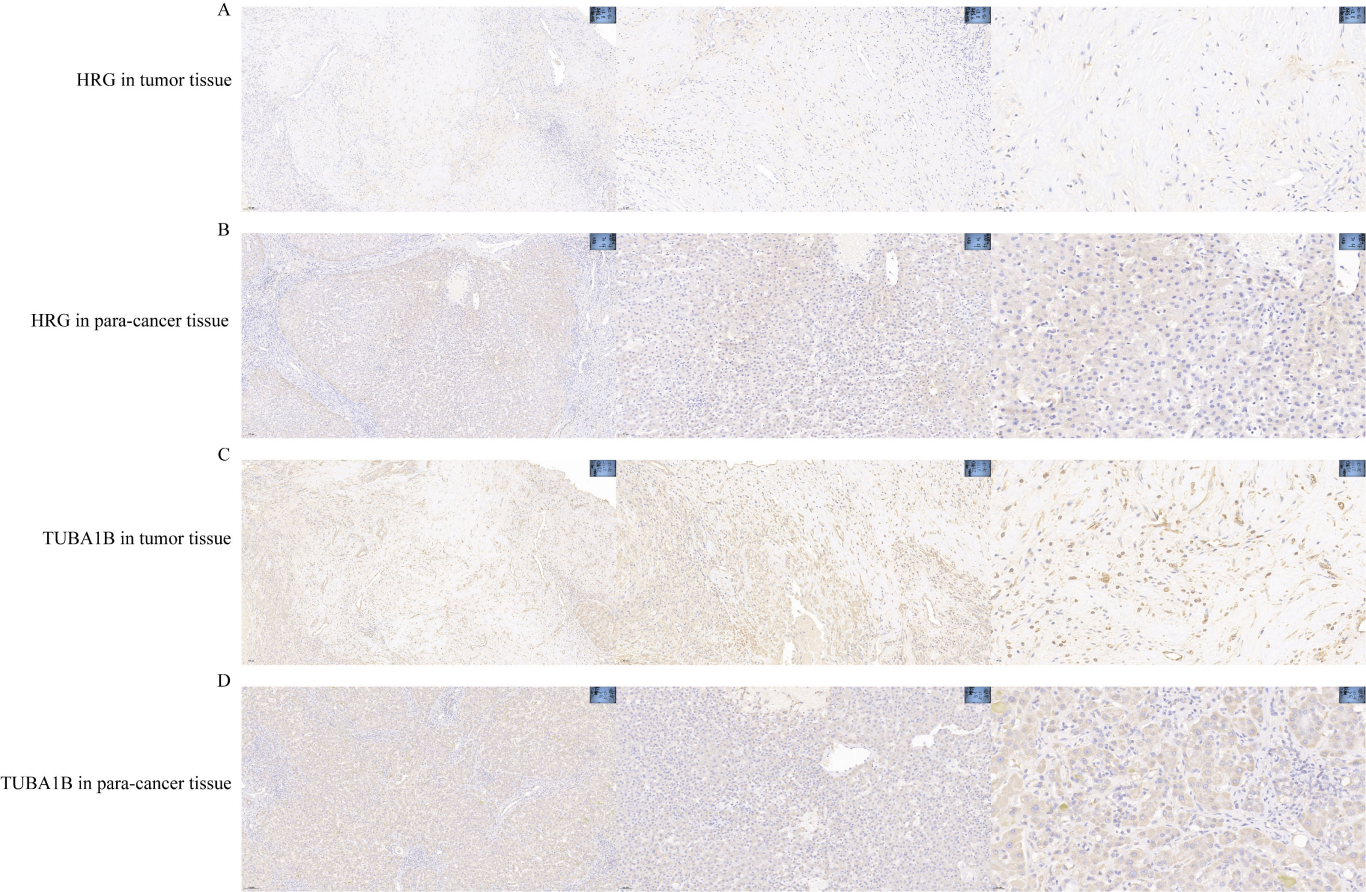
HRG gene and TUBA1B gene expression in HCC. **(A)** HRG gene in tumor tissue. **(B)** HRG gene in para-tumor tissue. **(C)** TUBA1B gene in tumor tissue. **(D)** TUBA1B gene in para-tumor tissue. HCC, hepatocellular carcinoma.

To validate the prognostic value of the prognostic genes, we used the IHC stain to detect the protein expression of HRG and TUBA1B in tumor tissues and normal tissues. The expression of HRG decreased in HCC compared with normal tissues, whereas TUBA1B expression levels increased in HCC compared with normal tissues (**Figure 7**).

## 4 Discussion

Hepatocellular carcinoma is highly heterogeneous, characterized by various morphologic features and biologic behaviors (35). Most patients with HCC are diagnosed at an advanced stage, which leads to rapid progress and poor outcomes due to the lack of effective and safe treatment (36, 37). Immune cells and stromal cells in TME are the main cellular components mediating immune tolerance and escape (38–43). Further studies on the heterogeneity of immune cells in the TME are indispensable for understanding their impact on prognosis. We developed a two-gene signature based on GEO (GSE148614) and TCGA-LIHC datasets and constructed a nomogram combining the gene signature and clinical features to predict OS in HCC.

Single-cell sequencing is a booming emerging technology in biomedical research and clinical practice, enabling comprehensive characterization of cell subpopulations, states, and lineages in heterogeneous tissues (44, 45). This is essential for the study of disease progression, tumor metastasis, response to treatment, and assessment of survival probability (25, 46). Therefore, scRNA has great development potential for promoting the diagnosis, targeted therapy, and prognosis prediction of cancers. We found that NK cell subsets were associated with prognosis based on scRNA-seq data from the GSE149614 dataset. NK cell infiltration is positively correlated with good prognosis (47, 48). Many studies have revealed that NK cell regulation of T cell function is an important immunomodulatory component in anticancer immunity (48–50). HCC patients with higher levels of intra-tumoral NK cell infiltration responded better to sorafenib treatment (51). These data strongly suggested that NK cell dysfunction contributes to HCC progression (52). In addition to the ability to kill malignant cells without prior sensitization, NK cells influence the activity of other immune cells by producing cytokines such as IFN-γ (53).

We applied consensus clustering on the TCGA-LIHC dataset, which can be effectively divided into 2 clusters, and the prognosis of the C1 and C2 clusters was significantly different. In WCGNA, we identified six modules and found that the turquoise module had the highest correlation with the prognostic cluster. The intersect was taken between NK cell genes and prognosis-related genes and yielded 25 genes. By univariate and multivariate Cox regression analysis, HRG and TUBA1B were correlated to survival outcomes of HCC. HRG is a secretory glycoprotein that binds to a variety of ligands, thereby regulating immunity, cell adhesion, angiogenesis, and thrombosis (54, 55). Inflammatory factors are pivotal in inflammatory diseases progression (56–60). HRG inhibits the activation of pro-inflammatory signaling (NF-κB) (61). Extensive studies have shown that the NF-κB signaling pathway is related to the development, progression, and invasion of tumors, and targeted regulation of the NF-κB signaling pathway can modulate these processes in various tumors. And NF-κB is bound to the inhibitory protein Farnesoid X (FXR), which retains NF–κB within the cytosol, thus preventing its transcriptional activity (62, 63). Additionally, HRG enhances the interaction between TNFR1 and Caspase8, promoting the formation of TNFR1 complex II, which directly induces apoptosis (64). Microtubules, which consist of a-tubulin and β-tubulin, perform important cellular functions such as protein trafficking, cell cycle, and cell migration (65). HRG also plays a significant role in modulating immune responses and angiogenesis, demonstrating potential in clinical applications for tumor targeting and sepsis-related immune regulation. Its immunomodulatory properties provide a foundation for developing novel therapeutic strategies, with promising applications in personalized treatments.

Microtubulin α1β (TUAB1B), an isoform of α-microtubulin, is associated with the expression of immune-related genes (66, 67). TUBA1B may play crucial roles in promoting tumor progression, including colon adenocarcinoma, osteosarcomas, liver hepatocellular carcinoma, and renal cell carcinoma (68–71). Furthermore, TUBA1B has been shown to mediate the infiltration of several immune cells in hepatocellular carcinoma and colorectal cancer. High TUBA1B expression is reported to be related to high paclitaxel resistance (72). Therefore, TUBA1B could also represent a therapeutic target for overcoming drug resistance, particularly in microtubule-targeting treatments, and holds promise for advancing precision oncology strategies. In our study, we further utilized IHC staining to assess the protein expression of HRG and TUBA1B in tumor and normal tissues. The results indicated that HRG expression was lower in HCC compared to normal tissues, while TUBA1B expression was higher in HCC, thereby confirming the prognostic value of these genes.

The identified genes have significant clinical applications in both immunotherapy and chemotherapy. In immunotherapy, these genes could help predict tumor response to treatment, guide the development of targeted therapies to enhance immune cell function, and enable personalized treatment strategies. As for chemotherapy, these genes can be used to predict drug resistance, assess therapeutic efficacy, and tailor chemotherapy regimens to individual patients, improving treatment outcomes and minimizing side effects. Additionally, the integration of therapies can be optimized by monitoring the expression of these genes, enabling real-time adjustments to treatment plans.

To assess the immune infiltration, ssGSEA analysis was performed. Based on the results of our study, the HRG gene and the TUBA1B gene can predict the prognosis of HCC and accurately respond to the tumor immune microenvironment. The HRG gene was correlated with anti-tumor immune cell infiltration, whereas TUBA1B gene was negatively correlated. The prognosis and immunotherapy outcomes are strongly influenced by the number of tumor-infiltrating lymphocytes (TILs) in the tumor microenvironment (73). Our findings provided new hints and references for the development of the immunotherapeutic approach for hepatocellular carcinoma.

There were limitations of the current study were acknowledged. Firstly, the sample size was small, and a prospective, large-sample, multicenter trial is warranted to confirm these findings. Secondly, functional studies on the molecular and biological functions of key genes will be required to substantiate this hypothesis. Thirdly, this study only identified NK cell-related genes, and further research is needed to explore other potentially related genes. Lastly, external validation using an independent cohort is required to assess the generalizability and reliability of our model.

Our study identified prognostically relevant NK cell signatures, which were further validated in clinical samples by immunohistochemistry. The findings had significant implications for the calculation of prognosis and therapeutic decision-making. Accurately assessing prognostic risk allows clinicians to identify individuals who are more likely to benefit from specific interventions or who require enhanced follow-up. Furthermore, discovering novel NK cell marker genes contributes to our understanding of the role of NK cells in HCC procession and provides a new strategy for precision immunotherapy.

## 5 Conclusion

In our study, we found a two-genes prognostic signature based on NK cell marker genes and elaborated on the role of the prognostic genes in the tumor immune microenvironment to provide new ideas for immunotherapy. In addition, we established an efficient and accurate prognostic model combined with clinical indicators to help clinicians make decisions.

## Data availability statement

The original contributions presented in the study are included in the article/**Supplementary Material**. Further inquiries can be directed to the corresponding authors.

## Ethics statement

The study had been approved by the Ethics Committee of Beijing Youan Hospital, Capital Medical University. As a minimal-risk study in compliance with the Helsinki protocol, the requirement for informed patient consent was waived by the same ethics committee that approved the study (Beijing Youan Hospital, Capital Medical University), and all methods were performed in accordance with relevant guidelines and regulations. In accordance with state law and institutional requirements, written informed consent for participation was not required in this study.

## Supplementary material

Supplementary Figure 1 Clustering and dimension reduction analysis of single cell data. **(A, B)** PCA and harmony were performed for dimension reduction analysis. **(C, D)** Gene numbers (nFeature RNA), sequencing depth (nCount RNA), and mitochondrial gene percentage (pMT) content of samples. **(E)** Correlation analysis of UMI and number of mRNA.

Supplementary Figure 2 Cell annotation of clusters was performed by classical markers of immune cells.

Supplementary Figure 3 Consensus clustering analysis from k=2 to k=9.

Supplementary Figure 4 Kaplan-Meier survival analysis of selected genes in normal and tumor samples. **(A)** Kaplan-Meier survival analysis of selected genes in TCGA cohort. **(B)** Kaplan-Meier survival analysis of selected genes in ICGC cohort. **(C)** HRG gene expression in relation to different tissues and clinical events. **(D)** TUBA1B gene expression in relation to different tissues and clinical events.

Supplementary Figure 5 Correlation analysis between the gene expression and immunity. **(A, B)** The relationship between HRG and TUBA1B expression with specific immune cell markers. **(C–E)** Correlation analysis between the gene expression and NK cells.

Supplementary Table 1 Top 20 marker genes of 27 clusters.

Supplementary Table 2 The differential genes obtained from TCGA cohort.
