# Supplementary figures and images for: Uncovering the heterogeneity of NK cells on the prognosis of HCC by integrating bulk and single-cell RNA-seq data
Source: Front Oncol. 2025 Mar 18;15:1570647. doi: 10.3389/fonc.2025.1570647 (PMC11959017; doi:10.3389/fonc.2025.1570647)

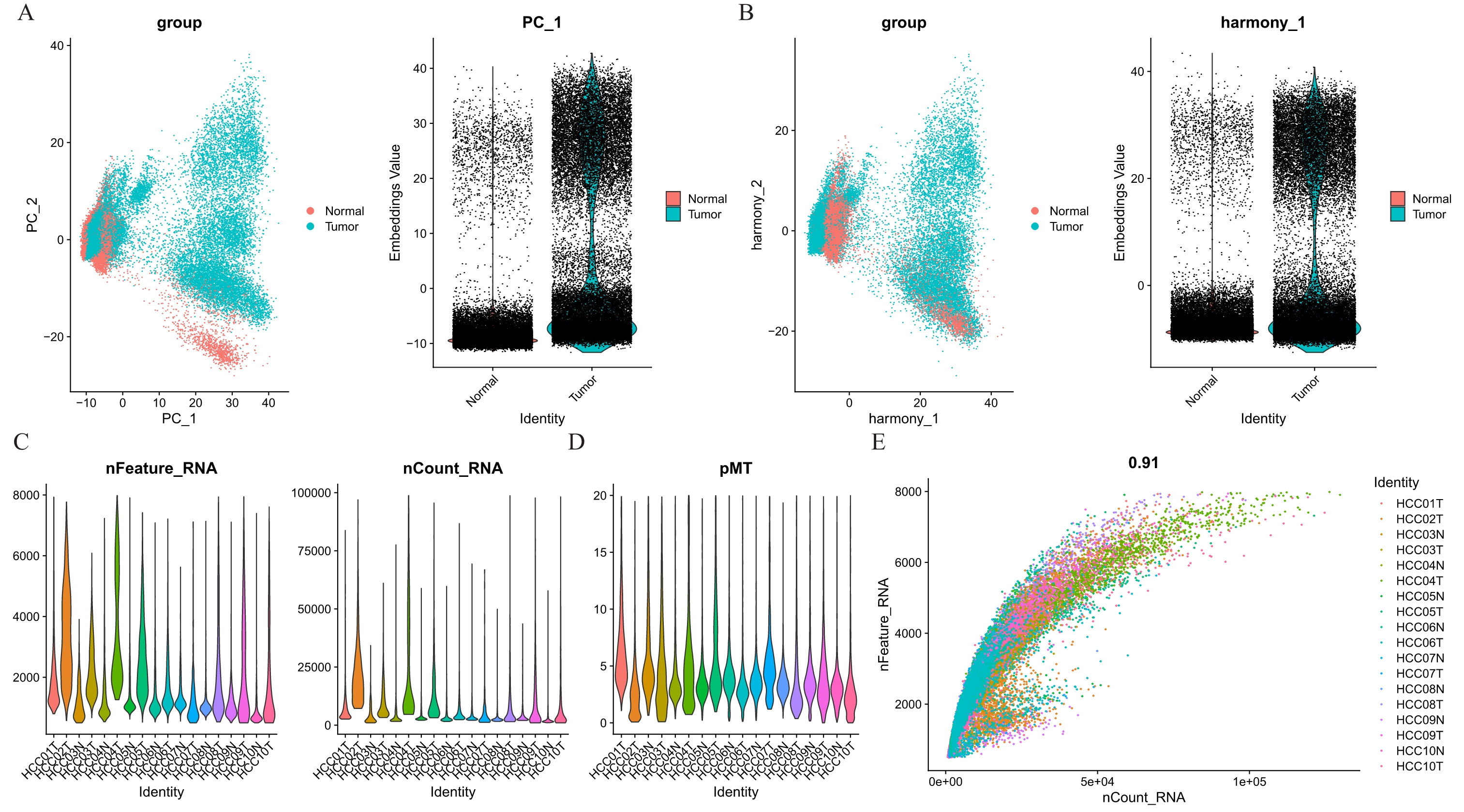

Supplement: Supplementary Figure 1 — Clustering and dimension reduction analysis of single cell data. (A, B) PCA and harmony were performed for dimension reduction analysis. (C, D) Gene numbers (nFeature RNA), sequencing depth (nCount RNA), and mitochondrial gene percentage (pMT) content of samples. (E) Correlation analysis of UMI and number of mRNA. [file Image1.jpeg]

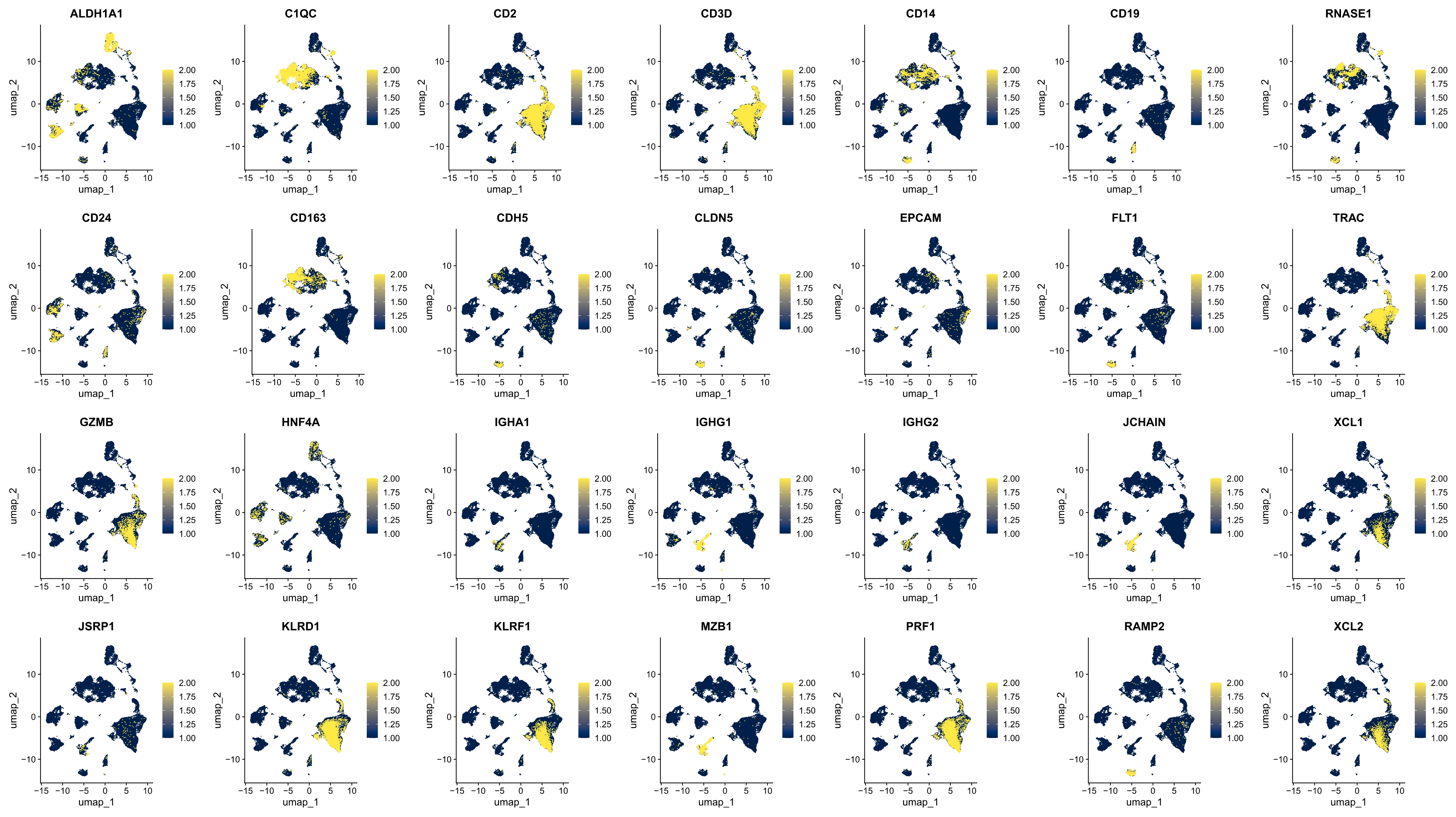

Supplement: Supplementary Figure 2 — Cell annotation of clusters was performed by classical markers of immune cells. [file Image2.jpeg]

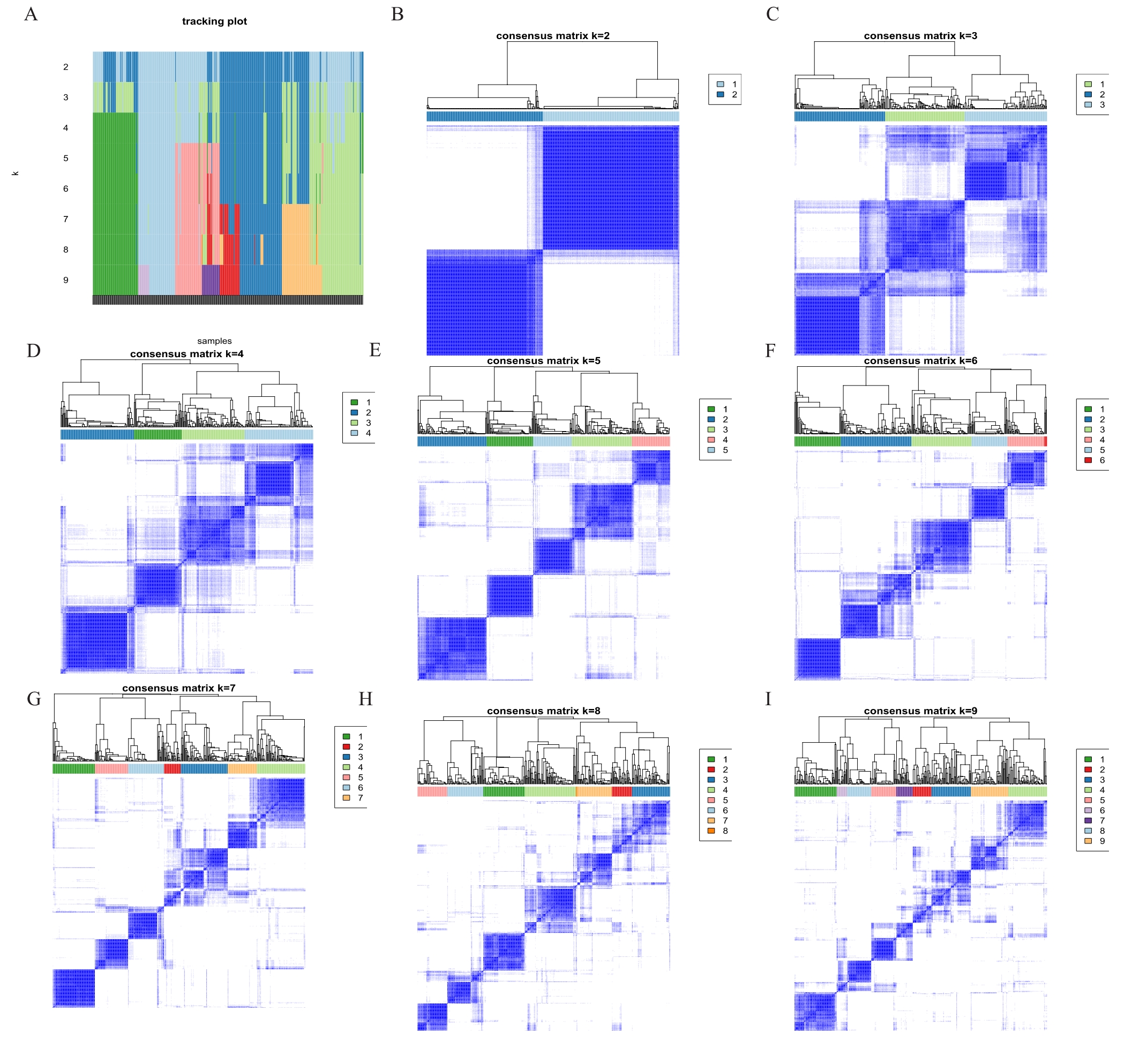

Supplement: Supplementary Figure 3 — Consensus clustering analysis from k=2 to k=9. [file Image3.jpeg]

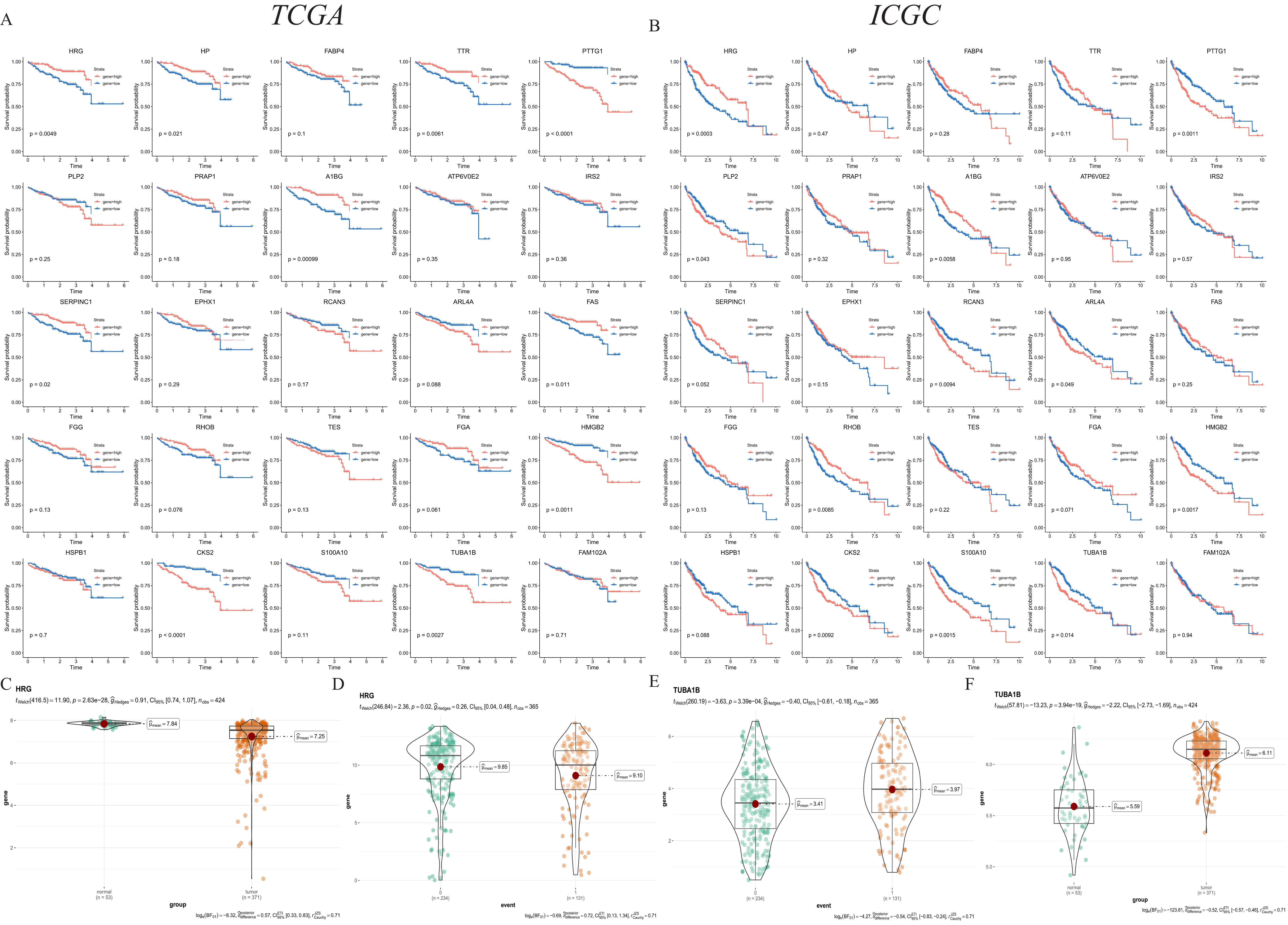

Supplement: Supplementary Figure 4 — Kaplan-Meier survival analysis of selected genes in normal and tumor samples. (A) Kaplan-Meier survival analysis of selected genes in TCGA cohort. (B) Kaplan-Meier survival analysis of selected genes in ICGC cohort. (C) HRG gene expression in relation to different tissues and clinical events. (D) TUBA1B gene expression in relation to different tissues and clinical events. [file Image4.jpeg]

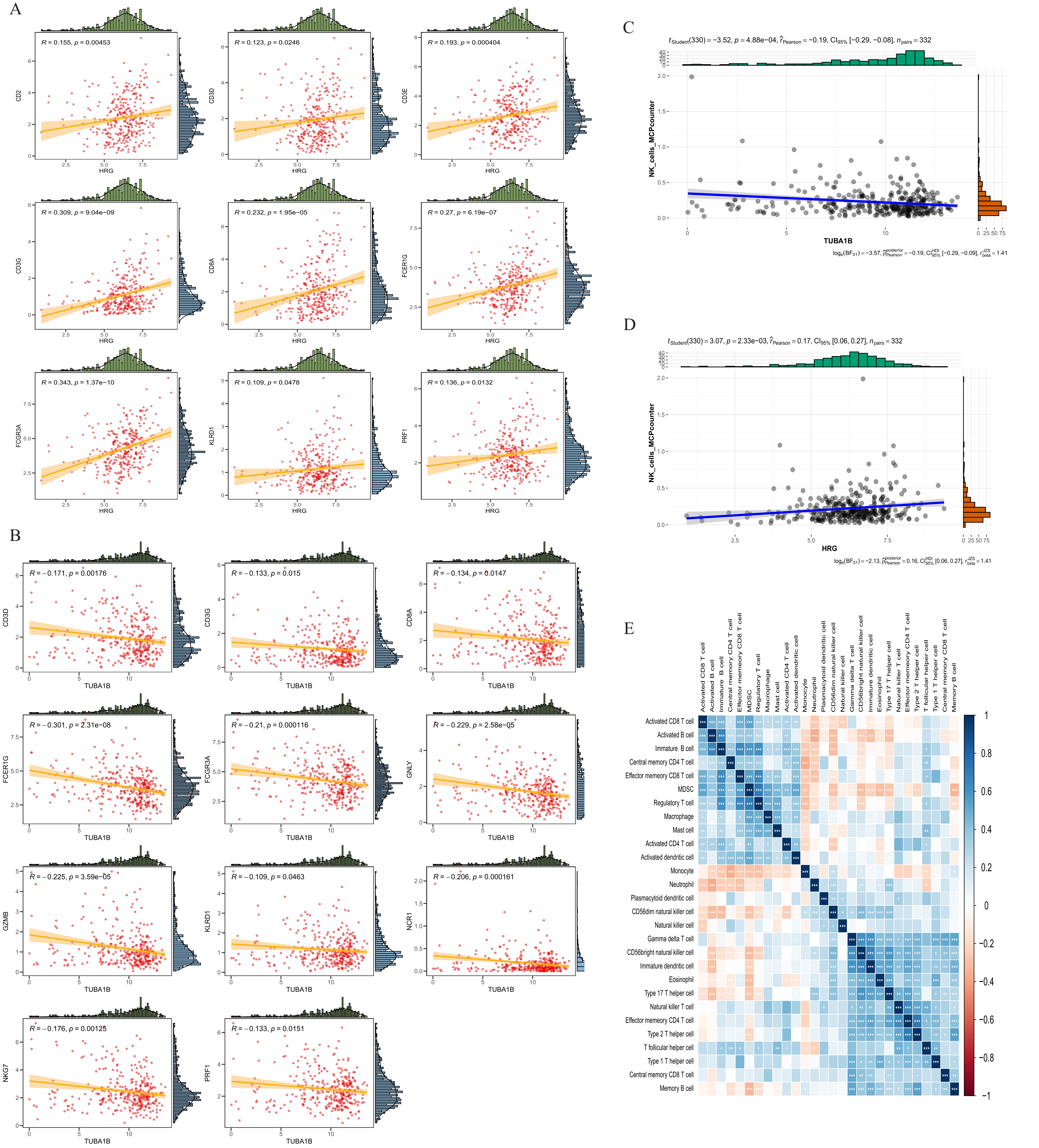

Supplement: Supplementary Figure 5 — Correlation analysis between the gene expression and immunity. (A, B) The relationship between HRG and TUBA1B expression with specific immune cell markers. (C–E) Correlation analysis between the gene expression and NK cells. [file Image5.jpeg]
